# The synergic effect of vincristine and vorinostat in leukemia *in vitro* and *in vivo*

**DOI:** 10.1186/s13045-015-0176-7

**Published:** 2015-07-10

**Authors:** Min-Wu Chao, Mei-Jung Lai, Jing-Ping Liou, Ya-Ling Chang, Jing-Chi Wang, Shiow-Lin Pan, Che-Ming Teng

**Affiliations:** Pharmacological Institute, College of Medicine, National Taiwan University, No. 1, Jen-Ai Road, Sec. 1, Taipei, Taiwan; Center for Translational Medicine, Taipei Medical University, No. 250, Wu-hsing Street, Taipei, 11031 Taiwan; School of Pharmacy, College of Pharmacy, Taipei Medical University, No. 250, Wu-hsing Street, Taipei, 11031 Taiwan; The Ph.D. Program for Cancer Biology and Drug Discovery, College of Medical Science and Technology, Taipei Medical University, No. 250, Wu-hsing St., Taipei, 11031 Taiwan

**Keywords:** Vincristine, SAHA, HDAC6, Leukemia

## Abstract

**Background:**

Combination therapy is a key strategy for minimizing drug resistance, a common problem in cancer therapy. The microtubule-depolymerizing agent vincristine is widely used in the treatment of acute leukemia. In order to decrease toxicity and chemoresistance of vincristine, this study will investigate the effects of combination vincristine and vorinostat (suberoylanilide hydroxamic acid (SAHA)), a pan-histone deacetylase inhibitor, on human acute T cell lymphoblastic leukemia cells.

**Methods:**

Cell viability experiments were determined by 3-[4,5-dimethylthiazol-2-yl]-2,5-diphenyltetrazolium bromide (MTT) assay, and cell cycle distributions as well as mitochondria membrane potential were analyzed by flow cytometry. *In vitro* tubulin polymerization assay was used to test tubulin assembly, and immunofluorescence analysis was performed to detect microtubule distribution and morphology. *In vivo* effect of the combination was evaluated by a MOLT-4 xenograft model. Statistical analysis was assessed by Bonferroni’s *t* test.

**Results:**

Cell viability showed that the combination of vincristine and SAHA exhibited greater cytotoxicity with an IC_50_ value of 0.88 nM, compared to each drug alone, 3.3 and 840 nM. This combination synergically induced G_2_/M arrest, followed by an increase in cell number at the sub-G_1_ phase and caspase activation. Moreover, the results of vincristine combined with an HDAC6 inhibitor (tubastatin A), which acetylated α-tubulin, were consistent with the effects of vincristine/SAHA co-treatment, thus suggesting that SAHA may alter microtubule dynamics through HDAC6 inhibition.

**Conclusion:**

These findings indicate that the combination of vincristine and SAHA on T cell leukemic cells resulted in a change in microtubule dynamics contributing to M phase arrest followed by induction of the apoptotic pathway. These data suggest that the combination effect of vincristine/SAHA could have an important preclinical basis for future clinical trial testing.

**Electronic supplementary material:**

The online version of this article (doi:10.1186/s13045-015-0176-7) contains supplementary material, which is available to authorized users.

## Background

Microtubules, dynamically structured polymers of α-tubulin and β-tubulin, are required in many cellular processes, including vesicle and organelle transportation, cellular motility, cell shape maintenance, chromosome separation, and division [1]. Before cells divide, microtubule-like reticular structures spread in the cytosol. As the cell enters the M phase, however, the original reticular microtubules are remodeled and assembled into mitotic spindles, which connect with chromosomes and drive chromosome separation [2]. All of the microtubule processes described above are crucial for microtubule polymerization (stabilization) and depolymerization (destabilization).

Drugs that inhibit microtubule dynamics have been used clinically in cancer treatment for more than 20 years [3]. These agents can be divided into two major types: microtubule-destabilizing agents, such as vincristine, and microtubule-stabilizing agents, such as paclitaxel [4]. At lower concentrations, both types affect only microtubule dynamics, inducing abnormal mitotic spindle formation and causing cell arrest in the M phase and, subsequently, cell apoptosis [5]. Microtubule-binding agents have played critical roles in the history of chemotherapeutic drug development and remain a first choice in the treatment of solid tumors and hematological malignancies. As with other anticancer agents, however, the problems of drug resistance, neuropathy, immunosuppression, and poor solubility must be addressed. The current trends in tubulin-binding agent development are to change the dosage to improve solubility or a combination of these agents with other anticancer drugs to reduce toxicity and enhance activity [6].

Histone deacetylase (HDAC) catalyzes the removal of the acetyl group in histones and nonhistone proteins. HDACs have different biological functions due to the highly divergent sequences outside the catalytic domain [7]. HDAC6, which predominates in the cytoplasm [8], is responsible for tubulin deacetylation, which plays a key regulatory role in the stability of the dynamic microtubules [9–12]. HDAC overexpression in many cancers has also been reported [13–15]. Thus, HDAC is believed to be a promising target for cancer therapy [16]. HDAC inhibitors (HDACis) cause histone hyperacetylation, thus activating the silenced gene for normal cellular function (cell cycle, angiogenesis, immunoregulation, cell apoptosis) [17]. In addition, HDACis have been found to target nonhistone proteins, including protein 53 (p53), E2F1, signal transducer and activator of transcription 1, nuclear factor-κβ, α-tubulin, heat shock protein 90, and Ku 70 [18–20]. Suberoylanilide hydroxamic acid (SAHA), a pan-HDACi, was the first HDACi approved by the FDA for cutaneous T cell lymphoma in 2006 [21–23]. Owing to their low toxicity and wide range of activity, HDACis are often combined with other anticancer drugs [24]. Most studies have been performed using cell-based experiments [23], but many combination therapies are undergoing clinical trials, often for hematological malignancies [17].

The purpose of this study is to evaluate the combined effects of vincristine and SAHA on an acute T cell lymphoblastic leukemia (ALL) model. We determined the mechanism underlying cell arrest in the mitotic phase and subsequent apoptosis following combination treatment. We found that vincristine and SAHA have a synergic effect on microtubules through different mechanisms. Moreover, this combined influence was also observed in *in vivo* results. Consequently, we suggest that a vincristine and SAHA combination treatment could be used in the clinical setting.

## Results

### Cytotoxic effects of vincristine and SAHA, alone and in combination, on human leukemic MOLT-4 cells

**Fig. 1:**
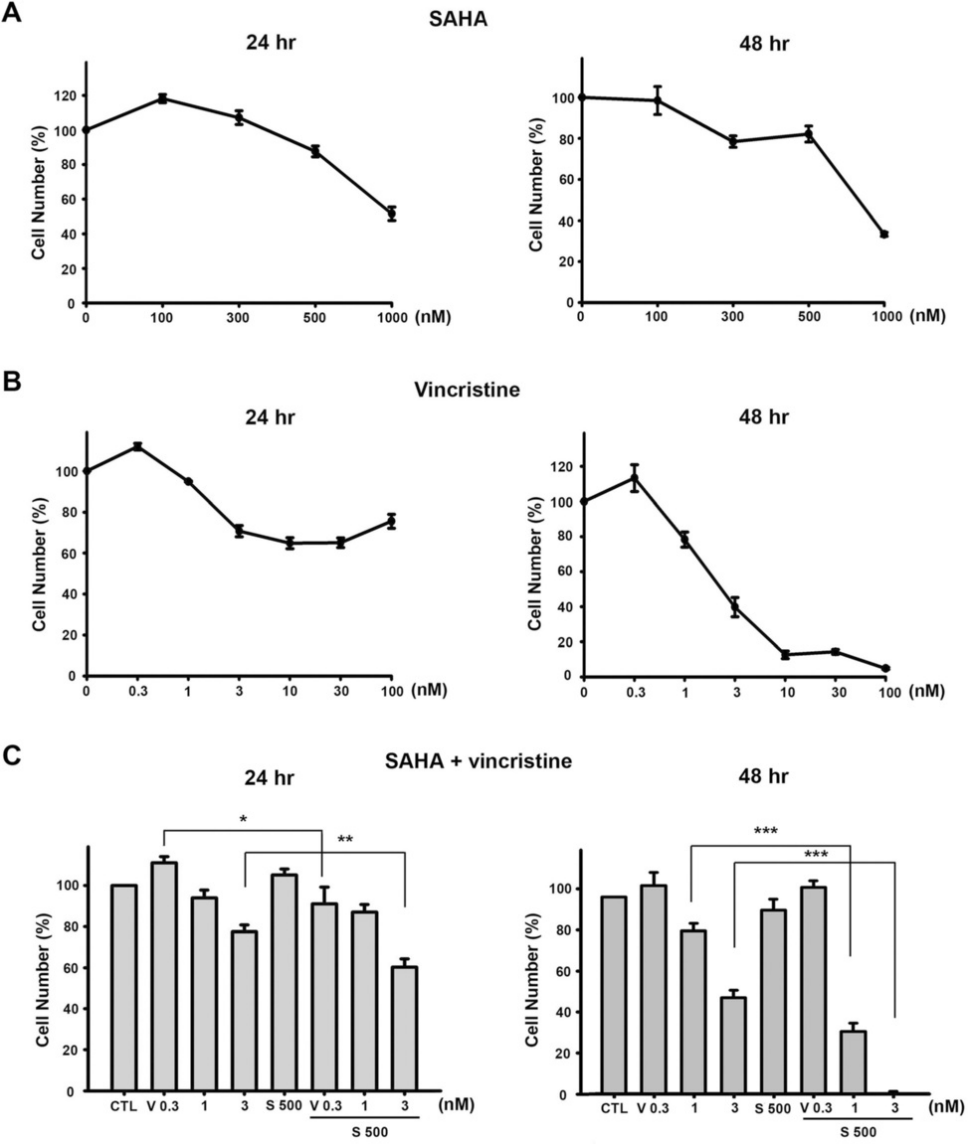
Cell viability effects of SAHA or vincristine alone and in combination on the MOLT-4 cell line. Cell viability was measured by MTT assay. **a**, **b** MOLT-4 cells were treated with various concentrations of SAHA and vincristine alone for 24 and 48 h, respectively. **c** The combination of SAHA (*S*) and vincristine (*V*) was compared to the effect of vincristine alone. Data are expressed as mean values ± SD of at least three separate determinations. *P < 0.05, ** *P* < 0.02, *** *P* < 0.005

A 3-[4,5-dimethylthiazol-2-yl]-2,5-diphenyltetrazolium bromide (MTT) assay was performed to investigate the cytotoxicity of the microtubule-destabilizing agent vincristine and the HDACi vorinostat (SAHA) on human ALL MOLT-4 cells. We first tested the cytotoxic effect of SAHA and vincristine alone and in combination. As shown in Fig. 1a, there was no significant cytotoxicity at concentrations up to 500 nM of SAHA. However, SAHA had an IC_50_ of 840 nM for 48 h, when concentration reached the highest level (1000 nM). In addition, vincristine exhibited cytotoxicity against human leukemic MOLT-4 cells with an IC_50_ of 3.3 nM at 48 h (Fig. 1b). To determine whether an interaction between SAHA and vincristine took place, the cytotoxic potency of a combination assay was measured. Cells treated with 500 nM SAHA and various concentrations of vincristine (0.3 to 3 nM) significantly inhibited cell survival compared to each treatment alone (Fig. 1c).

### Effects of vincristine in combination with SAHA on human T cell leukemic cell survival

**Fig. 2:**
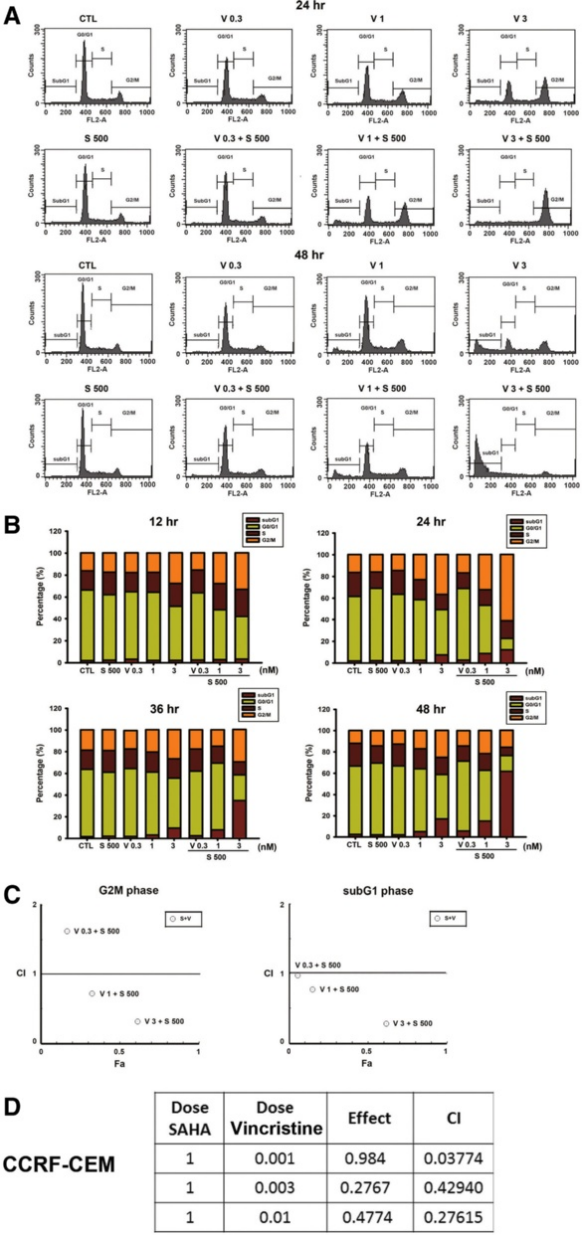
The combination of vincristine and SAHA had synergic effects on cell cycle kinetic changes. **a** MOLT-4 cells were treated with various concentrations of vincristine alone or in combination with SAHA (500 nM) for 24 and 48 h. **b** The quantitative data are shown in the time course. Cell cycle distribution was performed by cell cytometry after propidium iodide staining. **c** The combination effect of SAHA (500 nM) and vincristine (0.3, 1, and 3 nM) on G_2_/M arrest (*left figure*) and apoptosis (*right figure*) were used by the combination index (CI). And the CI was analyzed with the program CompuSyn (Ting-Chao Chou and Nick Martin). A CI value <1 represents that two drugs have synergic effects. **d** CCRF-CEM cells were treated with the indicated concentrations of vincristine alone or in combination with SAHA (1000 nM) for 48 h. The effect of sub-G_1_ phase was analyzed by flow cytometry. Data are expressed as mean values ± SD of at least three separate determinations. *Fa* fraction affected, *S* SAHA, *V* vincristine

To further explore the synergistic cytotoxic effects, we determined the effects on cell cycle distribution. As compared with SAHA, treatment with vincristine induced an increase in the G_2_/M phase of the cell cycle. In particular, the combination of vincristine plus SAHA caused an almost complete arrest of cells in the G_2_/M phase following short-term treatment (24 h) and a subsequent induction in the sub-G_1_ phase following long-term treatment (48 h) (Fig. 2a). Figure 2b shows the statistical results. Next, the combination index (CI) method was used to evaluate the synergistic combinations [25]. A CI value of >1.0, 1.0, and <1.0 indicates an antagonistic, additive, or synergistic interaction, respectively, between the drugs. In the G_2_/M phase, the CI values of vincristine (0.3, 1, and 3 nM) combined with 500 nM SAHA were 1.63, 0.72, and 0.32, respectively, and the CI values in the sub-G_1_ phase were 0.97, 0.77, and 0.28, respectively (Fig. 2c). And this synergistic combination effect also was noted in the other T cell leukemic cell line, CCRF-CEM (Fig. 2d), rather than in acute myeloid leukemic cells (Additional file 1: Figure S2). Moreover, vincristine (1 or 3 nM) combined with various concentrations of SAHA also shows synergistic effect (Additional file 2: Figure S1). These data indicate that vincristine and SAHA synergistically induced cell arrest in the G_2_/M phase and subsequently in the sub-G_1_ phase.

### Effects of SAHA in combination with vincristine on mitotic arrest in human leukemic MOLT-4 cells

**Fig. 3:**
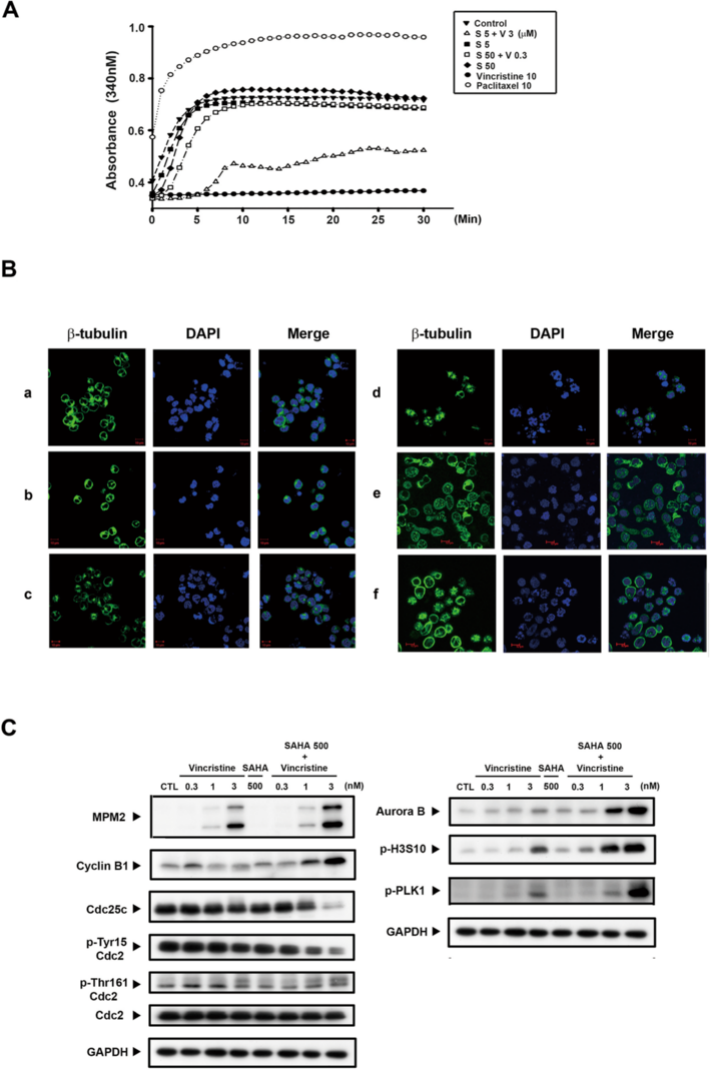
SAHA-sensitized MOLT-4 cells to vincristine-mediated mitotic arrest. **a** Tubulin assembly was determined by an *in vitro* tubulin polymerization kit and then detected by spectrophotometry. Paclitaxel (10 μM) and vincristine (10 μM) were used as positive controls. Paclitaxel is a polymerizing agent and vincristine is a depolymerizing agent. *S 5* represents SAHA 5 μM, and *S 50 + V 3* represents SAHA 50 μM combined with vincristine 3 μM. This experiment was examined in a cell-free condition. **b** MOLT-4 cells were treated with SAHA and vincristine alone or co-treatment for 24 h and then stained with β-tubulin and DAPI. The immunofluorescence images were captured by the ZEISS LSM 510 META confocal microscope. All images were under × 400 microscopic magnification. *a* control, *b* 500 nM SAHA, *c* 3 nM vincristine, *d* SAHA combined with vincristine, *e* 30 nM vincristine, *f* 30 nM paclitaxel. **c** Cells were treated with vincristine (0.3, 1, and 3 nM) alone or co-treated with SAHA (500 nM) for 24 h. Then, total cell lysates were obtained to evaluate the G_2_/M phase regulatory protein expression and the tubulin acetylation. Data are expressed as mean values ± SD of at least three separate determinations. *S* SAHA, *V* vincristine

To further elucidate the synergistic effect mechanism on the G_2_/M phase of cell cycle progression, we investigated SAHA in combination with vincristine on tubulin polarization change and mitosis-related proteins. As shown in Fig. 3a, there were no obvious tubulin polarization changes following SAHA treatment under cell-free conditions. However, in combination with vincristine, a significant induction of microtubule depolymerization was observed (Fig. 3a). Additional file 3: Figure S3 shows a more comprehensive result, including various vincristine- and SAHA-alone *in vitro* tubulin polymerization assays. To understand the effects of microtubule dynamics on mitosis following drug treatment, the microtubule arrangement in human leukemic MOLT-4 cells was examined by β-tubulin staining. As shown in Fig. 3b(b), there was no significant change in microtubule distribution and cell morphology after SAHA treatment. In addition, at low vincristine concentrations, cells had accumulated at the metaphase stage of mitosis with abnormal spindles (Fig. 3b(c)). In this study, spindles with bipolar and multipolar organization, which had abnormal long astral microtubules and chromosomes, were found to be unequally distributed. Nevertheless, at a high vincristine concentration, microtubule depolymerization was observed (Fig. 3b(d)). In the present study, the vincristine and SAHA combination exerted more explicit effects than vincristine alone with regard to abnormal spindles and chromosomes (Fig. 3b and Additional file 4: Figure S4). These results suggest that SAHA potentiated the effects of vincristine due to inhibition of microtubule dynamics.

M phase-regulating proteins were also analyzed. The combination of various concentrations of vincristine (0.3, 1, and 3 nM) with SAHA (500 nM) for 24 h increased the phosphorylation of two mitotic markers (MPM2 and H3S10) in a concentration-dependent manner. These results proved that the combination of vincristine and SAHA induced M phase arrest. Moreover, vincristine in combination with SAHA also induced mitotic arrest by stimulating cyclin B1, aurora B, phospho-Cdc2 (Thr161), and phospho-PLK1 expression, and suppressing Cdc25c and phospho-Cdc2 (Tyr15) levels. The magnitude of these changes was more obvious than those observed with vincristine treatment alone (Fig. 3c). These findings demonstrate that SAHA enhanced vincristine-induced M phase arrest and that the combination therapy induced microtubule dynamics instability and mitotic activation.

### Effects of SAHA in combination with vincristine on the apoptotic pathway and HDAC activity in human leukemic MOLT-4 cells

**Fig. 4:**
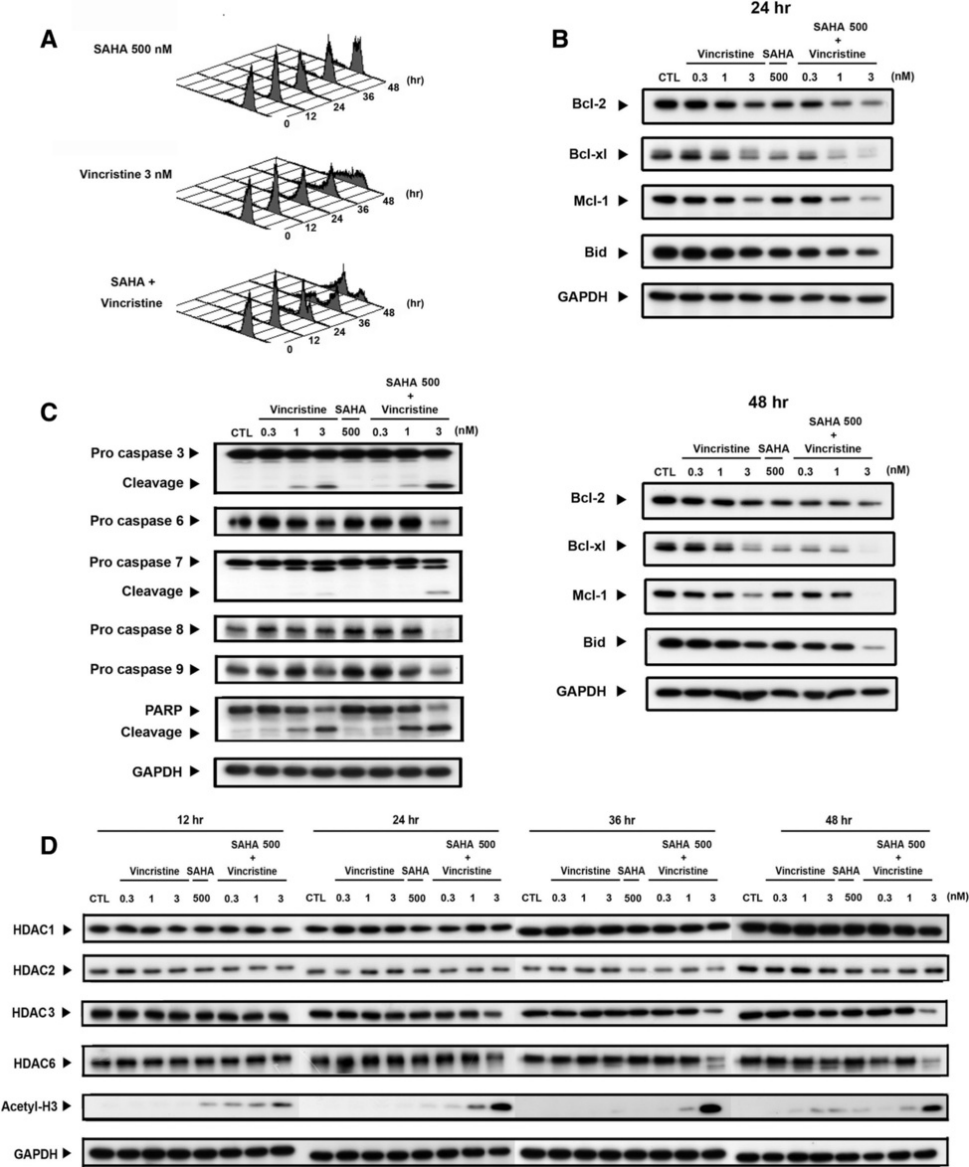
Co-treatment with vincristine and SAHA induced mitochondrial membrane potential loss, caspase activation, and HDAC activity. **a** The mitochondrial membrane potential was measured by the flow cytometry analysis of rhodamine 123. The MOLT-4 cells were stained with 10 μM rhodamine 123 and incubated at 37 °C for 30 min in the presence of SAHA alone (500 nM), vincristine alone (3 nM), or coexistence of both at different time intervals (12, 24, 36, and 48 h). The *horizontal axis* shows the relative fluorescence intensity and the *vertical axis* presents the cell number. **b** MOLT-4 cells were treated with different concentrations of vincristine (0.3, 1, and 3 nM) alone, a single concentration of SAHA (500 nM) alone, and a combination with both drugs for 24 and 48 h. Then, the cell lysates were collected for western blot analysis to detect the Bcl-2 family protein levels. **c** Cells were incubated with vincristine and SAHA for 48 h, and caspase 3, 6, 7, 8, and 9 activations and PARP cleavage were detected. **d** Similarly, the total cell lysates were analyzed to measure HDAC1, HDAC2, HDAC3, and HDAC6 expression and HDAC activity (H3 acetylation). Data are expressed as mean values ± SD of at least three separate determinations. *S* SAHA, *V* vincristine

Mitochondria play a crucial role both in the intrinsic and extrinsic apoptotic pathways. To test whether the vincristine/SAHA-mediated apoptotic pathway was associated with mitochondrial function, a change in mitochondrial transmembrane potential (Δ*ψ*m) was assessed. As shown in Fig. 4a, treatment with SAHA or vincristine alone was insufficient to affect the mitochondrial membrane potential; however, this phenomenon was enhanced by co-treatment with SAHA in a time-dependent manner. The Bcl-2 protein family plays a regulatory role in controlling the mitochondrial apoptotic pathway. The data showed that the combination treatment more effectively downregulated the expression of the pro-survival members of the Bcl-2 family, such as Bcl-2, Bcl-xl, and Mcl-1, than did either treatment alone (Fig. 4b).

Caspase activation plays an important role in the classic apoptotic pathways. In this study, it was also found that caspases 3, 6, 7, 8, and 9 and PARP were activated by the combination treatment for 48 h (Fig. 4c). Moreover, to confirm whether HDACs, both in the single or combination treatment, were involved in the apoptosis pathway, the expression of HDACs and H3 acetylation were evaluated. Vincristine (3 nM) in combination with SAHA effectively inhibited HDAC3 and HDAC6 expression and enhanced H3 acetylation, which represents an inhibition in HDAC activity, in a time-dependent manner (Fig. 4d).

### HDAC6 inhibition was involved in vincristine/SAHA-induced apoptosis

**Fig. 5:**
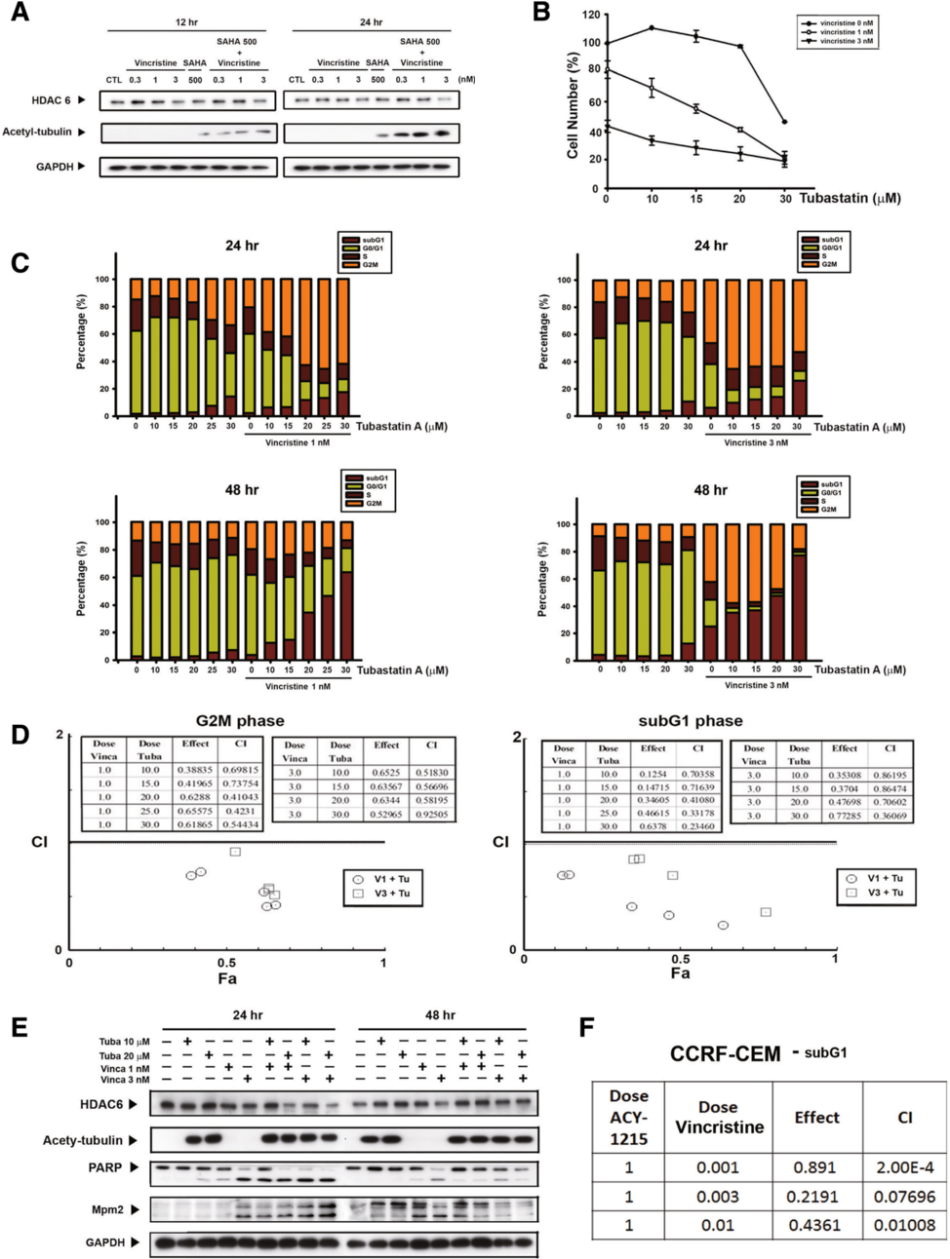
HDAC6 was involved in vincristine/SAHA-induced cell death. **a** MOLT-4 cells were treated with the indicated drugs for 12 and 24 h. The cell lysates were used to determine the HADC6 protein level and activity. **b** Cells were treated with tubastatin A alone, which is a specific HDAC6 inhibitor, or combined with 1 and 3 nM vincristine for 48 h. The cell viability was evaluated by MTT assay. **c** Cells incubated with tubastatin A alone or in combination with the indicated concentration of vincristine for 24 and 48 h. The cell cycle distribution was measured by flow cytometry. The figures are shown as quantitative data in the time course. **d** The CI value of combining tubastatin A with vincristine on G_2_/M and sub-G_1_ phases. **e** Cells were treated with 10 or 20 μM tubastatin A (*Tuba*) alone and combined with 1 or 3 nM vincristine. The cell lysates were used for western bolt analysis. **f** CCRF-CEM cells were co-treated with vincristine and ACY1215, an HDAC6-specific inhibitor, for 48 h. The CI value of combining treatment on the sub-G_1_ phase

Previous findings have shown HDAC6-induced tubulin acetylation to affect the dynamics and function of microtubules [9–12]. As shown in Fig. 5a, SAHA, a pan-HDACi, induced tubulin acetylation; however, its combination with vincristine had no synergic effect. Tubastatin A, which is a specific HDAC6 inhibitor [26], was used to understand the role of HDAC6 in the vincristine/SAHA-treated cells. To evaluate the potential benefit of vincristine in combination with tubastatin A, the cytotoxicity of co-treatment was determined and the combination effects were analyzed. However, compared to tubastatin A alone, vincristine significantly enhanced the cytotoxicity of tubastatin A (Fig. 5b). Moreover, vincristine (1 and 3 nM) combined with various concentrations of tubastatin A induced cell accumulation at the G_2_/M phase followed by the sub-G_1_ phase (Fig. 5c). The CI values were <1 in combination of vincristine and tubastatin at the G_2_/M phase and sub-G_1_ phase (Fig. 5d). Co-treatment of vincristine and tubastatin revealed MPM2 and PARP activation consistent with the induction of apoptosis by western blot analysis (Fig. 5e). And vincristine and HDAC6 inhibitor combined synergism effect was further corroborated by the observation of vincristine and ACY1215 co-treatment in CCRF-CEM cells (Fig. 5f). These findings suggest that SAHA treatment may alter microtubule dynamics in cells through HDAC6 inhibition, even though the effect was insufficient to arrest cells in the G_2_/M phase. However, in combination with vincristine, which also had an effect on microtubules, SAHA caused extreme microtubule stress thus causing cell death.

### The antitumor activity of vincristine and SAHA combination therapy *in vivo*

**Fig. 6:**
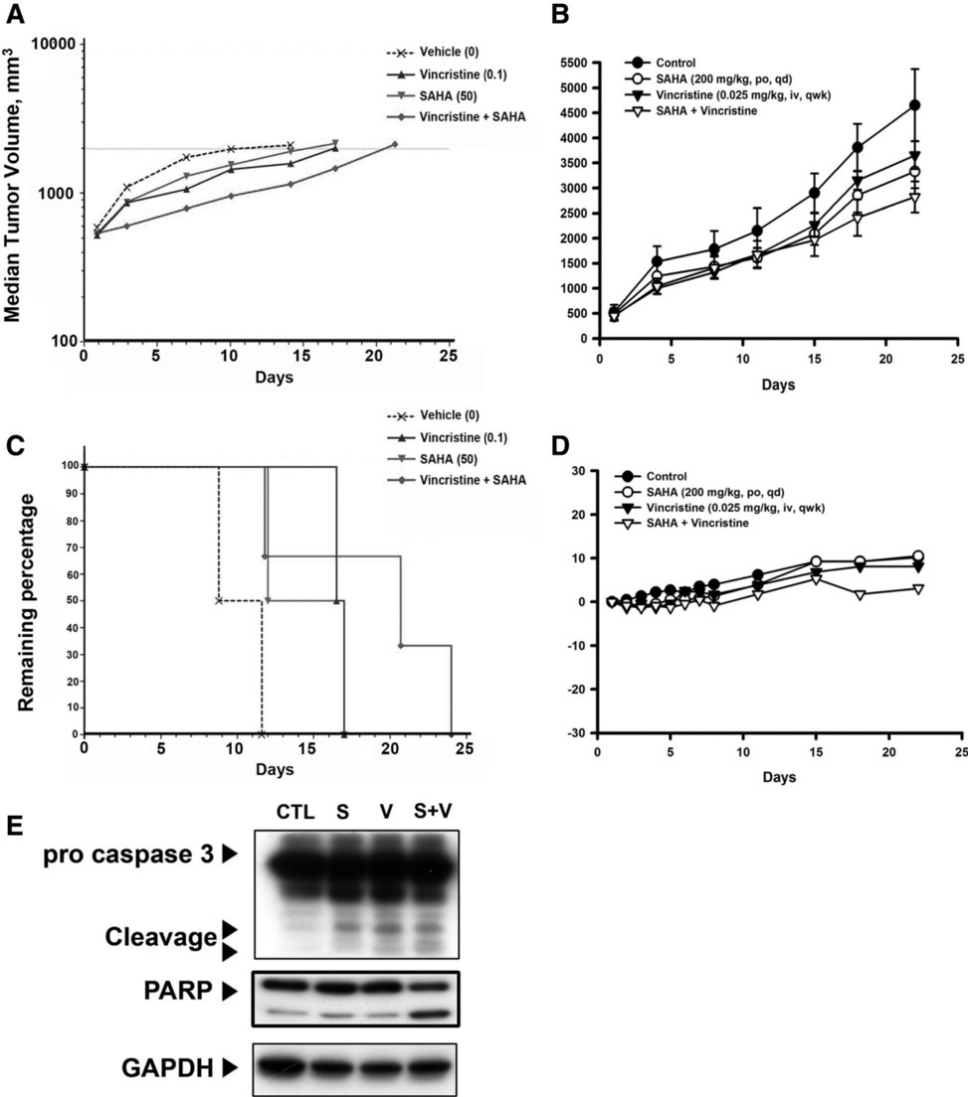
The effect of vincristine and SAHA on the MOLT-4 tumor xenograft. SCID mice were ectopically implanted with MOLT-4 cells, and when the tumor size reached 100 mm^3^, mice were injected with vincristine (i.p., qwk) or SAHA (p.o., qd) alone or a combination of both. **a** *In vivo* antitumor efficacy was expressed as tumor growth delay (TGD) (vincristine 0.1 mg/kg i.v. qwk; SAHA 50 mg/kg p.o. qd). **b** Effects of combination on tumor volume. The *growth curves* are the means of the tumor sizes measured with each group (vincristine 0.025 mg/kg i.v. qwk; SAHA 200 mg/kg p.o. qd). **c** The remaining percentage of mice survival (vincristine 0.1 mg/kg i.v. qwk; SAHA 50 mg/kg p.o. qd). **d** Effects of combined treatment on mice body weight (vincristine 0.025 mg/kg i.v. qwk; SAHA 200 mg/kg p.o. qd). **e** Tumors were resected for the western blot analysis (vincristine 0.025 mg/kg i.v. qwk; SAHA 200 mg/kg p.o. qd)

To evaluate whether the synergistic effect of vincristine plus SAHA could be clinically relevant, the antitumor activity of this co-treatment in severe combined immunodeficiency mice bearing established MOLT-4 tumor xenografts was investigated. Once a tumor was palpable (approximately 100 mm^3^), mice were randomized into vehicle control and treatment groups (*n* = 6 per group). All mouse tumors were allowed to reach an endpoint volume of 2000 mm^3^, and *in vivo* antitumor efficacy was expressed as tumor growth delay (TGD; Fig. 6a). There were no improvements in TGD in mice treated with vincristine (0.1 mg/kg once weekly) or SAHA (50 mg/kg once daily) alone. However, log-rank analysis showed that the co-treatment exhibited significant antitumor activity in the MOLT-4 xenograft model (*P* = 0.0389). In addition, Kaplan–Meier curves displayed antitumor activity for the co-treatment group (vincristine, 0.025 mg/kg once weekly; SAHA, 200 mg/kg once daily) (Fig. 6b). Notably, the mice tolerated all of the treatments without overt signs of toxicity; no significant body weight difference or other adverse side effects were observed (Fig. 6d and Additional file 5: Figure S5). To correlate the *in vivo* antitumor effects with the mechanisms identified *in vitro*, intratumoral biomarkers were assessed by western blot analysis. Consistent with *in vitro* results, the combined treatment markedly induced caspase 3 activation and PARP cleavage in tumors, indicating elevated apoptosis (Fig. 6e). Taken together, these findings suggest that combination of vincristine and SAHA, both *in vitro* and *in vivo*, dramatically enhanced vincristine-induced cell death.

## Discussion

Recent preclinical studies have reported that due to their broad anticancer potency and low toxicity, HDACis are often used in combination therapy to enhance conventional chemotherapeutic and molecular-targeted drugs. SAHA was the first HDACi approved by the FDA for T cell lymphoma. Moreover, vinca alkaloids have been extensively used in the clinical treatment of ALL. In spite of their usefulness, drug resistance and neuron toxicity remain serious clinical problems. Therefore, the purpose of this study was to investigate the anticancer activity of vincristine and SAHA in a T cell ALL cell line. Cytotoxic experiments have shown that the combination of vincristine and SAHA significantly induces cell death. Analyses of cell cycle and regulatory protein expressions have indicated that co-treatment with the two drugs has a synergistic effect on M phase arrest and is consistent with an increase of cell numbers in the sub-G_1_ phase. Furthermore, combination treatment promotes cell apoptosis through intrinsic and extrinsic pathways. *In vivo* xenograft animal models demonstrated that, compared to treatment with either drug alone, vincristine in combination with SAHA prolongs survival time in mice, as suggested by the *in vitro* results.

During these studies, the level of the effects of vincristine and SAHA on MOLT-4 cells was determined. Combinations with various concentrations of both drugs were investigated to determine the lowest effective concentration of vincristine or SAHA that would provide the maximum cytotoxic effects. It was established that the cytotoxicity of 3 nM vincristine combined with 500 nM SAHA is much more potent in inhibiting cell survival (Fig. 1) and altering cell cycle distribution (Fig. 2) compared to either treatment alone. A CI value smaller than 1 at the G_2_/M or sub-G_1_ phases suggests that the combination of vincristine and SAHA had a synergistic action on T cell leukemic cells at certain concentrations (Fig. 2).

Prior studies have shown that α-tubulin acetylation is associated with microtubule dynamics and is regulated by HDAC6 [27]. Additional research has also found that HDACis induce microtubule acetylation and polymerization through HDAC6 inhibition [9–12]. Whether the polymerization or the depolarization of microtubules following the vincristine and SAHA combination treatment was responsible for G_2_/M cell cycle arrest needs to be determined for a complete understanding of this mechanism. *In vitro* tubulin polymerization assays showed that a high concentration of vincristine (10 μM) definitely caused microtubule depolymerization, but a high concentration of SAHA (50 μM) had no influence on microtubule polymerization (Fig. 3a). However, SAHA indeed causes the acetylation of tubulin (Figs. 4d and 5a). These results suggest that SAHA may induce tubulin acetylation, subsequently affecting microtubule polymerization by inhibiting HDAC6 activity. Nevertheless, SAHA-induced polymerization was not proven (Fig. 3a) since HDAC6 was not available in the *in vitro* tubulin polymerization kits, which contained only tubulin and GTP. Therefore, no SAHA-induced polymerization effects were observed in this assay. In addition, the literature indicates that tubulin acetylated by certain HDACis, such as trichostatin A, is associated with microtubule dynamics without affecting the polymerization of microtubules. Therefore, to further demonstrate the role of HDAC6, tubastatin A, a specific HDAC6 inhibitor, was used [26–28]. Tubastatin A and vincristine co-treatment was found to synergically induce cell death and G_2_/M phase cell arrest, proceeding to the sub-G_1_ phase, compared with tubastatin A or vincristine alone (Fig. 5). These phenomena were in agreement with the results of vincristine/SAHA combination treatment. Consequently, an immunofluorescence assay was performed to demonstrate whether microtubules were affected by SAHA. Figure 3b shows that at low vincristine concentrations, abnormal spindles (star-like monopolar and multiple polars) and chromosome disorganization were observed, as has been previously reported by Jordan et al. [26]. However, the microtubule distribution remained unchanged following cell treatment with 500 nM SAHA. However, following SAHA/vincristine combination treatment, more cells were observed to have deteriorated spindles although without microtubule depolymerization or polymerization. Prior studies have shown that only higher concentrations (>10 nM) of microtubule agents, such as vinca alkaloids and taxol, affect microtubule mass, but microtubule dynamics are suppressed by low microtubule agent concentrations (<10 nM) [4]. Taken together, vincristine/SAHA-induced synergistic G_2_/M arrest may result from HDAC6 inhibition-induced microtubule dynamic alternation because of SAHA as well as vincristine. In brief, vincristine and SAHA exerted a synergic action on microtubule dynamics, albeit though different mechanisms.

A large amount of research shows that HDACis arrest cells in the G_1_ phase mainly through p21 induction. Only a few studies mention the role of these compounds in G_2_/M, and the mechanism remains uncertain. For example, Blagosklonny et al. found that the HDACi trichostatin A causes tubulin acetylation that contributes to M phase arrest [28]. HDACis have also shown the capability to cause immature sister chromatid separation and slippage from the mitotic spindle assembly checkpoint (SAC) [29]. Moreover, cancer cells without wild-type p21 or p53 more easily become polyploid than wild-type cells [30]. These results indicate that HDACi-induced G_2_/M arrest is not due to an influence on transcriptional activity. In contrast, other studies point out that trichostatin A causes G_2_/M cell cycle arrest and SAC slippage through an increase in p21 transcriptional activity [31]. The mechanisms proposed above to explain vincristine/SAHA-induced G_2_/M phase arrest should not be excluded. We found that p21 mRNA and protein level were elevated after treatment with vincristine or SAHA alone and was not enhanced by combined treatment (data not shown). It has been reported that all HDACis induced p21 but differentially caused tubulin acetylation, mitotic arrest, and cytotoxicity. Mitotic arrest rather than induction of p21 determined HDACi cytotoxicity [28]. Moreover, p21 is required for G_1_ arrest, not for cell death, and is associated with resistance to HDACi-induced apoptosis [32]. Upregulated p21 was also found in vincristine-treated cells even though vincristine caused cell arrest in the G_2_/M phase, leading to apoptosis [33]. Therefore, we considered that in our study, increased p21 expression of vincristine-SAHA combined treatment was the cellular protection mechanism for repairing damaged DNA, and this phenomenon was not sufficient to cause apoptosis. Furthermore, vincristine and SAHA did not alter p53 protein and mRNA levels (data not shown). Consequently, the involvement of p53 and p21 in drug-induced G_2_/M arrest was eliminated.

The expression of related proteins in the M phase was evaluated to understand the exact phase at which the cells arrest (G_2_ or M). Cdc25c is a tyrosine phosphatase that removes the inhibitory phosphorylation of Cdc2 at Tyr15, which has already been phosphorylated on Thr161 and contributes to Cdc2 activation. The Cdc2/cyclin B complex then forms to move cells into the M phase [34]. Figure 3c shows that Cdc25c and p-Tyr15 Cdc2 protein levels declined and p-Thr161 Cdc2 increased following combination treatment. In addition, expression of the M phase markers pMPM2 and H3S10 was also induced [35]. These results demonstrate that combination treatment induces cell arrest in the M phase and not in G_2_.

From the above data, we speculate that the vincristine/SAHA combination may alter microtubule dynamics, resulting in incorrect microtubule attachment to the centromere of chromosomes or loss of spindle tension across kinetochore pairs, subsequently causing SAC activation in the metaphase. An increased aurora B and PLK kinase protein expression and aurora B kinase activity, detected by H3S10 expression downstream of aurora B, are shown in Fig. 3c. SAC activation inhibits anaphase-promoting complex/cytochrome activity and decreases cyclin B degradation to inter-anaphase to accomplish cell division. Cells proceed to apoptosis owing to an inability to repair the dysfunction [36]. Therefore, we suggest that co-treatment-induced cell death was due to an inability to repair microtubule function even if the SAC was activated (Figs. 3c and 4c). In contrast, Dowling et al. have found that the synergistic effect of the HDACi trichostatin A and microtubule agents occurs through SAC inactivation [37]. In addition, studies have shown that HDACis induce aurora B kinase degradation and subsequently inactivate the SAC via HDAC3 inhibition [38]. Although HDAC3 is inhibited by 24-h vincristine and SAHA co-treatment (Fig. 4d), the protein level of aurora B kinase was increased (Fig. 3c). We believe that the arrest at the G_2_/M phase does not occur through the inhibition of HDAC3.

Caspase activation plays a vital role in the apoptosis pathways. Figure 4c shows that the activation of caspases 3 and 6 to 9 and PARP was synergistically induced by vincristine combined with SAHA. This result indicates that the combination treatment activates both the intrinsic and the extrinsic apoptosis pathways. Therefore, the expression of mitochondrial proteins and mitochondrial membrane potential were investigated. Bid is a key protein linking the extrinsic and intrinsic apoptosis pathways. As Fig. 4b shows, the protein level of pro-form Bid decreased in response to co-treatment with vincristine and SAHA. Decreases in pro-survival mitochondrial proteins Bcl-2, Bcl-xl, and Mcl-1 also occurred. A decrease in mitochondrial membrane potential was significantly induced by the combination in a time-dependent manner.

Taking all of the above findings into consideration, when vincristine and SAHA treatments were combined, MOLT-4 cell survival was significantly inhibited, mainly through the induction of M stage arrest and intrinsic and extrinsic apoptosis pathways. Moreover, the selective HDAC6 inhibitor exhibited similar synergism when combined with vincristine. *In vivo* xenograft animal models produced the same results as those of *in vitro* models. Pan-HDAC inhibitors influenced different types of HDACs, which caused side effects more than selective HDAC6 inhibitors. However, the most important and practical advantage to choose SAHA for this study is that SAHA has been approved for cancer treatment. Selective HDAC6 inhibitors such as tubastatin A and ACY1215 are only in phase I or phase II clinical trial. The finding of this paper not only provides another choice for clinical treatment but also offers an idea for the development and future application of HDAC6 inhibitors.

## Methods

### Cell lines

The human T cell acute lymphoblastic leukemic cell lines, MOLT-4 and CCRF-CEM, isolated from the relapsed and multiresistant patients, were obtained from Bioresource Collection and Research Center (Taiwan). Cells were maintained in RPMI-1640 medium supplemented with 10 % (*v*/*v*) fetal bovine serum (Gibco BRL Life Technologies, Grand Island, NY, USA) and 1 % of a mixture of penicillin (100 U/ml) and streptomycin (100 μg/ml, Biological Industries Ltd., Kibbutz Beit Haemek, Israel). All cells were cultured in an incubator in the presence of 5 % CO_2_ at 37 °C.

### Chemicals and antibodies

Vincristine and suberoylanilide hydroxamic acid (SAHA) were purchased from Sigma Chemical Co. (St. Louis, MO, USA), and tubastatin A (HDAC6 inhibitor) was synthesized from Dr. Jing-Ping Liou (Taipei Medical University, Taiwan). The above drugs were dissolved in DMSO (dimethylsulfoxide) and then preserved at −20 °C. Rhodamine 123, 3-(4,5-dimethylthiazol-2-yl)-2,5-diphenyltetrazolium, propidium iodide, anti-β-tubulin, FITC-conjugated anti-mouse IgG, and all of the other chemical reagents used in this study were purchased from Sigma Chemical (St. Louis, MO, USA) and of analytical grade. Primary antibodies against Cdc2 (pY15), aurora B, caspase 8, caspase 9, HDAC1, HDAC2, HDAC3, HDAC4, and Bid were all purchased from Cell Signaling Technology (Beverly, MA); cyclin B, Cdc25C, Cdc2, PARP, Mcl-1, Bcl-2, Bcl-xl, and secondary antibodies were purchased from Santa Cruz (Santa Cruz, CA, USA); MPM2 (pSer/Thr) and H3 (pS10) were purchased from Upstate Biotechnology (Lake Placid, NY, USA); PLK (pT210), caspase 6, and caspase 7 were purchased from BD Biosciences (San Jose, CA, USA); caspase 3 was purchased from Imgenex (San Diego, CA, USA); acetyl-histone 3 was purchase from Millipore (Billerica, MA, USA); and an internal control, GAPDH, was purchased from Novus Biologicals (Littleton, CO, USA). Vectashield® mounting medium with DAPI was purchased from Burlingame, CA, USA.

### Cell viability assay

Cell viability was verified by MTT assay. Firstly, cells were seeded in a 24-well plate at a density of 4 × 10^5^ cells/well in 1 ml culture medium and then treated with various concentrations of vincristine or SAHA alone or a combination of both for 24 and 48 h. After treatment with drugs, 100 μl MTT solution (0.5 mg/ml in phosphate-buffered saline (PBS)) per well was added to the 24-well plate in the dark and the plate was incubated at 37 °C. The mitochondrial dehydrogenase of viable cells reduced MTT (yellow) to insoluble formazan dyes (purple). One hour later, the crystal formazan dyes were dissolved in the extraction buffer (0.1 M sodium acetate buffer, 100 μl/well). The absorbance was spectrophotometrically analyzed at 550 nm by an ELISA reader (Packard, Meriden, CT, USA).

### Flow cytometry analysis

Evolution of the cell cycle histogram was performed by flow cytometry analysis to detect the changes in DNA content. Cells (1 × 10^6^) were seeded in a 6-well plate in 2 ml fresh medium and treated with graded concentrations of vincristine, SAHA, or combination for the indicated time. Then, cells were collected, washed with PBS and fixed with 70 % (*v*/*v*) ice cold ethanol at −20 °C for 30 min. The fixed cells were centrifuged to remove the ethanol, rinsed with PBS, resuspended in 0.1 ml DNA extraction buffer (0.2 M Na_2_HPO_4_-0.1 M citric buffer, pH 7.8) for 20 min, and subsequently stained with 500 μl PI solution (80 μg/ml propidium iodide, 100 μg/ml RNase A, and 1 % Triton X-100 in PBS) for 20 min at room temperature in the dark. Data were analyzed by FACScan Flow Cytometer and CellQuest software (Becton Dickinson).

### *In vitro* tubulin polymerization assay

To determine the microtubule polymerization of the indicated drugs in a cell-free condition, CytoDYNAMIX Screen 03 Kit (Cytoskeleton Inc.) was performed. General tubulin buffer, GTP stock (100 mM), and tubulin protein (10 mg/ml) were all prepared well following the protocol. A 96-well plate was placed in the spectrophotometer to prewarm at 37 °C for 30 min before detection. Then preparing the iced tubulin polymerization (TP) buffer, all mentioned processes were needed on the ice. Next, the drugs (2 μl) were added into each Eppendorf included with 85 μl TP buffer. The drugs must include DMSO (the control group), paclitaxel (10 μΜ), and vincristine (10 μΜ). Paclitaxel and vincristine were used as positive controls. Paclitaxel would induce the microtubule polymerization; in contrast, vincristine would depolymerize the microtubules. Finally, 30 μl tubulin proteins was added into the Eppendorf and transferred to the prewarmed 96-well plate. The absorbance was measured by a spectrophotometer and recorded every 1 min for 30 min at 340 nm and 37 °C.

### Immunofluorescence analysis

Microtubule distribution and morphology were detected by immunofluorescence. Cover slides were placed in the 24-well plate and coated with poly-d-lysine for 1 day at least to enhance the suspension cells attached to the cover slides. Cells were seeded into the 24-well plate (8 × 10^5^ cells/well) and treated with vincristine, SAHA, or both drugs for 24 h. The following experiments were performed at room temperature. The cells were fixed with 8 % paraformaldehyde in PBS for 15 min. After washing with PBS for several times, the cells were permeabilized with 0.1 % Triton X-100 in PBS for 10 min. Then, the cells were rinsed with PBS for 10 min three times. For blocking, 3 % BSA in PBS was used. After 1 h, the cells were washed with PBS and incubated with a primary β-tubulin antibody (1:200) for 2 h and FITC-conjugated anti-mouse IgG antibody (1:200) for 2 h. The mounting medium, which contains DAPI stain, was dropped onto the slides, and cover slides were recovered to the slides. Images were detected and captured with the ZEISS LSM 510 META confocal microscope.

### Western blot analysis

After the treatment, cells (10^6^ cells/ml) were harvested. Whole cell pellets were washed twice with PBS, lysed in lysis buffer (50 mM Tris, pH 7.4; 150 mM NaCl; 1 % Triton X-100; 1 mM EDTA; 1 mM EGTA) supplemented with protease inhibitors (1 mM PMSF, 10 μg/ml aprotinin, 10 μg/ml leupeptin, 1 mM sodium orthovanadate, and 1 mM NaF) for 30 min, and then centrifuged for 30 min at 13,000 rpm at 4 °C. Total protein content was quantified by BCA Protein Assay Kit (Thermo Fisher Scientific, Rockford, IL, USA). Equivalent amounts of proteins were separated into diverse percentages by SDS-PAGE and subsequently transferred onto PVDF membranes. The membranes were blocked with 5 % nonfat milk in PBS for 1 h at room temperature and incubated with primary antibody in PBST buffer (0.1 % Tween 20 in PBS) at 4 °C overnight. After washing the membranes with PBST, blots were developed with the corresponding HRP-conjugated secondary antibody diluted in 0.5 % nonfat milk in PBS for 1 h at room temperature. The membrane was washed frequently with PBST, and finally immunoreactive protein bands were displayed using an enhanced chemiluminescence detection kit (Amersham, Buckinghamshire, UK).

### Mitochondrial membrane potential

Rhodamine 123 was used to evaluate mitochondrial membrane potential. Rhodamine 123 is a kind of cationic fluorescent dye, which localizes in the mitochondria. Loss of mitochondrial membrane potential is associated with a lack of rhodamine 123 retention and a decrease of fluorescence intensity. Cells were treated with vincristine, SAHA, or combination for the indicated time. Rhodamine 123 (final concentration 10 μM) was added and incubated for 30 min at 37 °C in the dark. Then, cells were harvested and rinsed with PBS. The fluorescence intensity was measured by FACScan Flow Cytometer and CellQuest software (Becton Dickinson).

### Tumor xenograft model

A tumor xenograft model was used to estimate the combination effect of vincristine and SAHA *in vivo*. MOLT-4 cells were implanted (10^7^ cells/ml) into severe combined immunodeficiency (SCID) mice. When the average tumor size reached 100 mm^3^, mice were treated with an indicated dosage of vincristine or SAHA alone or a combination of both. Mice were scarified until the average tumor size was larger than 2500 mm^3^. Then, tumors were resected and frozen for the western blot analysis to evaluate the effect of vincristine/SAHA combination *in vivo*. All animal experiments followed ethical standards, and protocols have been reviewed and approved by the Animal Use and Management Committee of National Taiwan University (IACUC Approval No.: 20100225).

### Statistical analysis

All experiments were done independently three times and presented as mean values ± SD, and then assessed by Bonferroni’s *t* test. The animal experiments were determined by the Mann-Whitney test. *P* < 0.05 was considered statistically significant.

## Additional files

Additional file 1: Figure S2. The effect of vincristine and SAHA co-treatment in acute myeloid leukemic cell lines. (A) HL60 (acute promyelocytic leukemia) cells were co-treated with the indicated vincristine and SAHA, and the CI values of the sub-G_1_ phase were analyzed. (B) The cell viability of U937 (acute monocytic leukemia) after the indicated treatment. V, vincristine; S, SAHA; Tuba, tubastatin A. Additional file 2: Figure S1. The effect of vincristine combined with various doses of SAHA. (A) MOLT-4 cells were treated with various SAHA alone or combined with 1 and 3 nM vincristine for 48 h. The cell viability was evaluated by MTT assay. (B) The distribution of cell cycle after MOLT-4 cells were treated with various concentrations of SAHA alone or in combination with vincristine 1 or 3 nM for 24 and 48 h. The quantitative data are shown in the time course. (C) The combination effect on G_2_/M arrest (left figure) and apoptosis (right figure) were used by the combination index (CI). Additional file 3: Figure S3. The results of vincristine- (0.3 and 3 μM), or SAHA (5 and 50 μM) alone, or combination of both *in vitro* tubulin polymerization assay. (A) The figure shows more comprehensive results on *in vitro* tubulin polymerization, including paclitaxel (10 μM), vincristine (0.3, 3, and 10 μM), SAHA (S, 5 and 10 μM) alone, and SAHA combined with vincristine (S 50 + V 0.3 and S 5 + V3). (B) The absorbance of different drugs at the endpoint (30 min). Additional file 4: Figure S4. The quantity of abnormal spindles in vincristine alone compared to combination with SAHA. MOLT-4 cells were treated with SAHA (0.5 μM) and vincristine alone (3 nM) or co-treatment for 24 h and then stained with β-tubulin and DAPI. The figure shows the quantity of abnormal spindles by the immunofluorescence images. Additional file 5: Figure S5. The cell toxicity of normal cells after combination treatment. The cell viability (MTT assay) of (A) HUVEC (human umbilical vein endothelial cell) and (B) BEAS-2B (human bronchial epithelial cell) after co-treating with the indicated drugs for 48 h.

## Abbreviations

ALL: acute lymphoblastic leukemia
HDAC: histone deacetylase
TGD: tumor growth delay
