# Supplementary figures and images for: The synergic effect of vincristine and vorinostat in leukemia in vitro and in vivo
Source: J Hematol Oncol. 2015 Jul 10;8:82. doi: 10.1186/s13045-015-0176-7 (PMC4504084; doi:10.1186/s13045-015-0176-7)

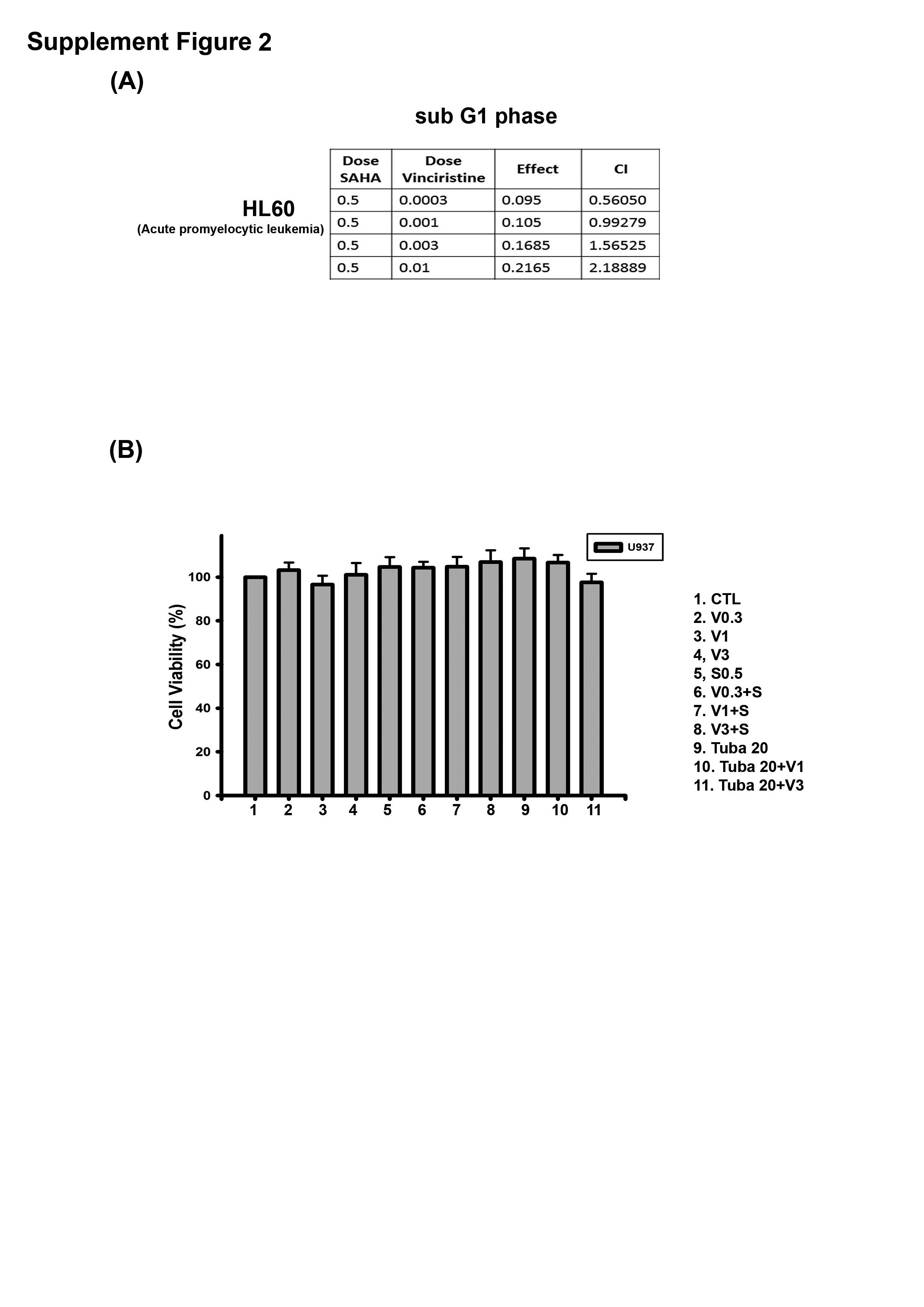

Supplement: Additional file 1: Figure S2. — The effect of vincristine and SAHA co-treatment in acute myeloid leukemic cell lines. (A) HL60 (acute promyelocytic leukemia) cells were co-treated with the indicated vincristine and SAHA, and the CI values of the sub-G1 phase were analyzed. (B) The cell viability of U937 (acute monocytic leukemia) after the indicated treatment. V, vincristine; S, SAHA; Tuba, tubastatin A. [file 13045_2015_176_MOESM1_ESM.jpg]

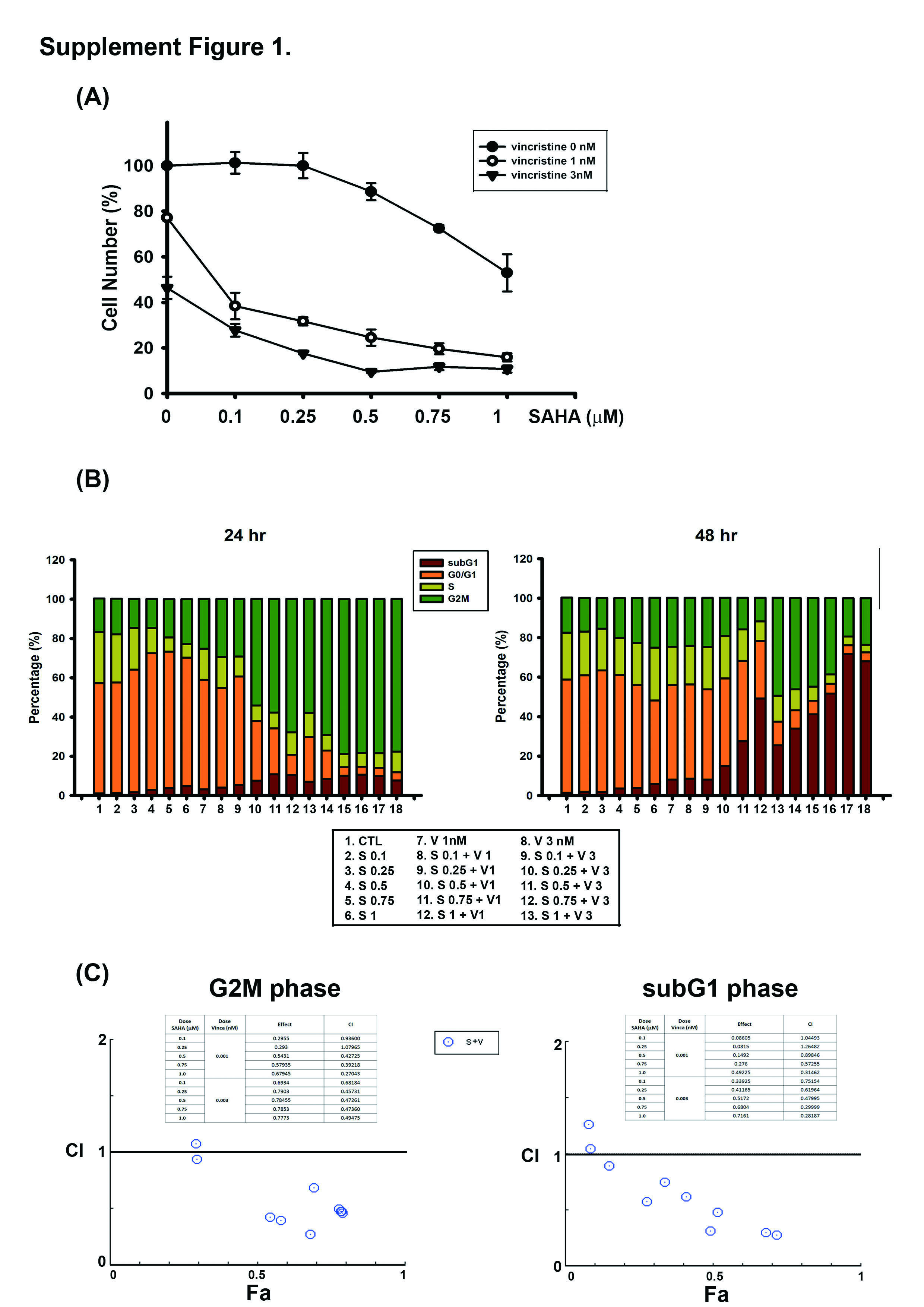

Supplement: Additional file 2: Figure S1. — The effect of vincristine combined with various doses of SAHA. (A) MOLT-4 cells were treated with various SAHA alone or combined with 1 and 3 nM vincristine for 48 h. The cell viability was evaluated by MTT assay. (B) The distribution of cell cycle after MOLT-4 cells were treated with various concentrations of SAHA alone or in combination with vincristine 1 or 3 nM for 24 and 48 h. The quantitative data are shown in the time course. (C) The combination effect on G2/M arrest (left figure) and apoptosis (right figure) were used by the combination index (CI). [file 13045_2015_176_MOESM2_ESM.jpg]

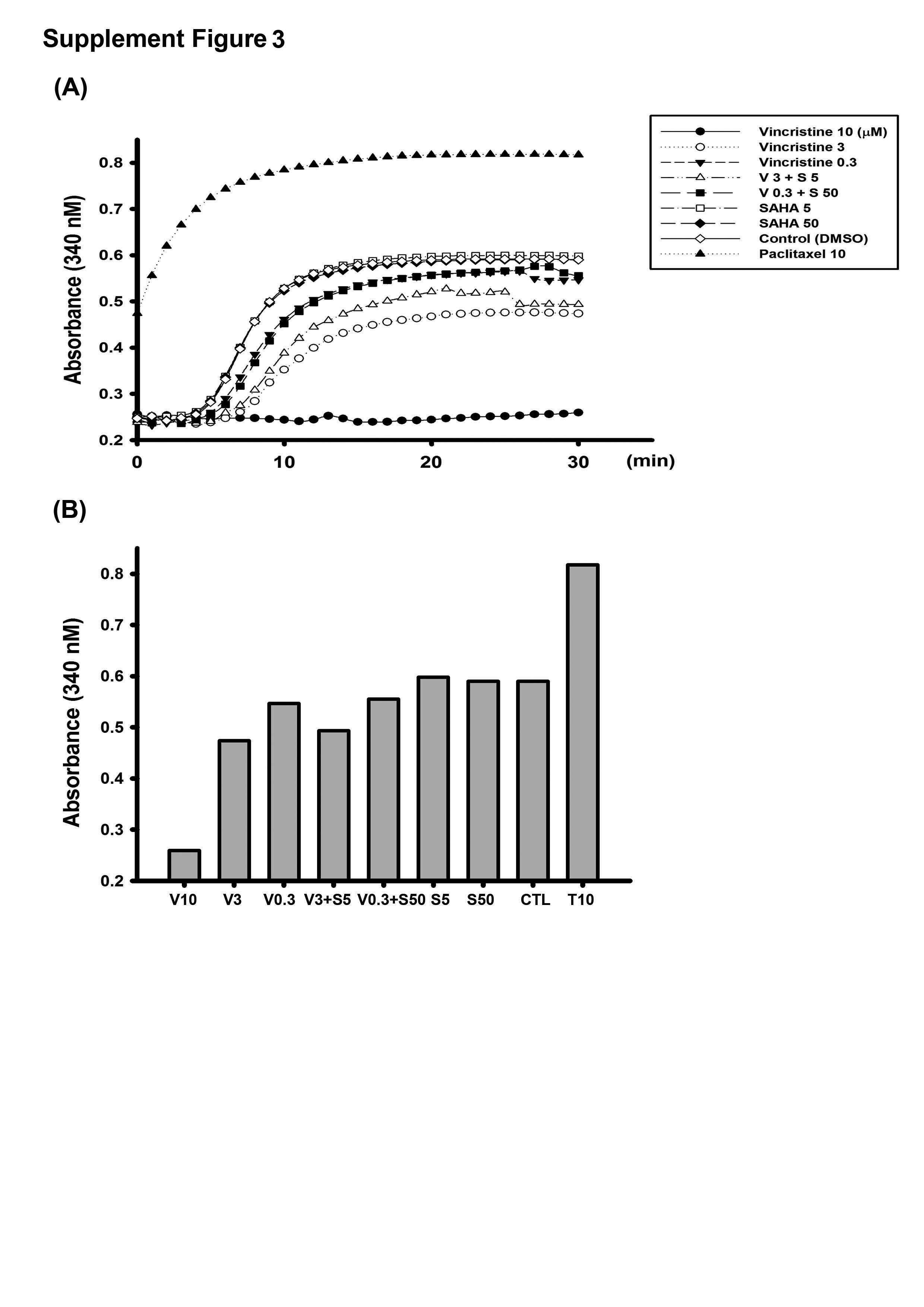

Supplement: Additional file 3: Figure S3. — The results of vincristine- (0.3 and 3 μM), or SAHA (5 and 50 μM) alone, or combination of both in vitro tubulin polymerization assay. (A) The figure shows more comprehensive results on in vitro tubulin polymerization, including paclitaxel (10 μM), vincristine (0.3, 3, and 10 μM), SAHA (S, 5 and 10 μM) alone, and SAHA combined with vincristine (S 50 + V 0.3 and S 5 + V3). (B) The absorbance of different drugs at the endpoint (30 min). [file 13045_2015_176_MOESM3_ESM.jpg]

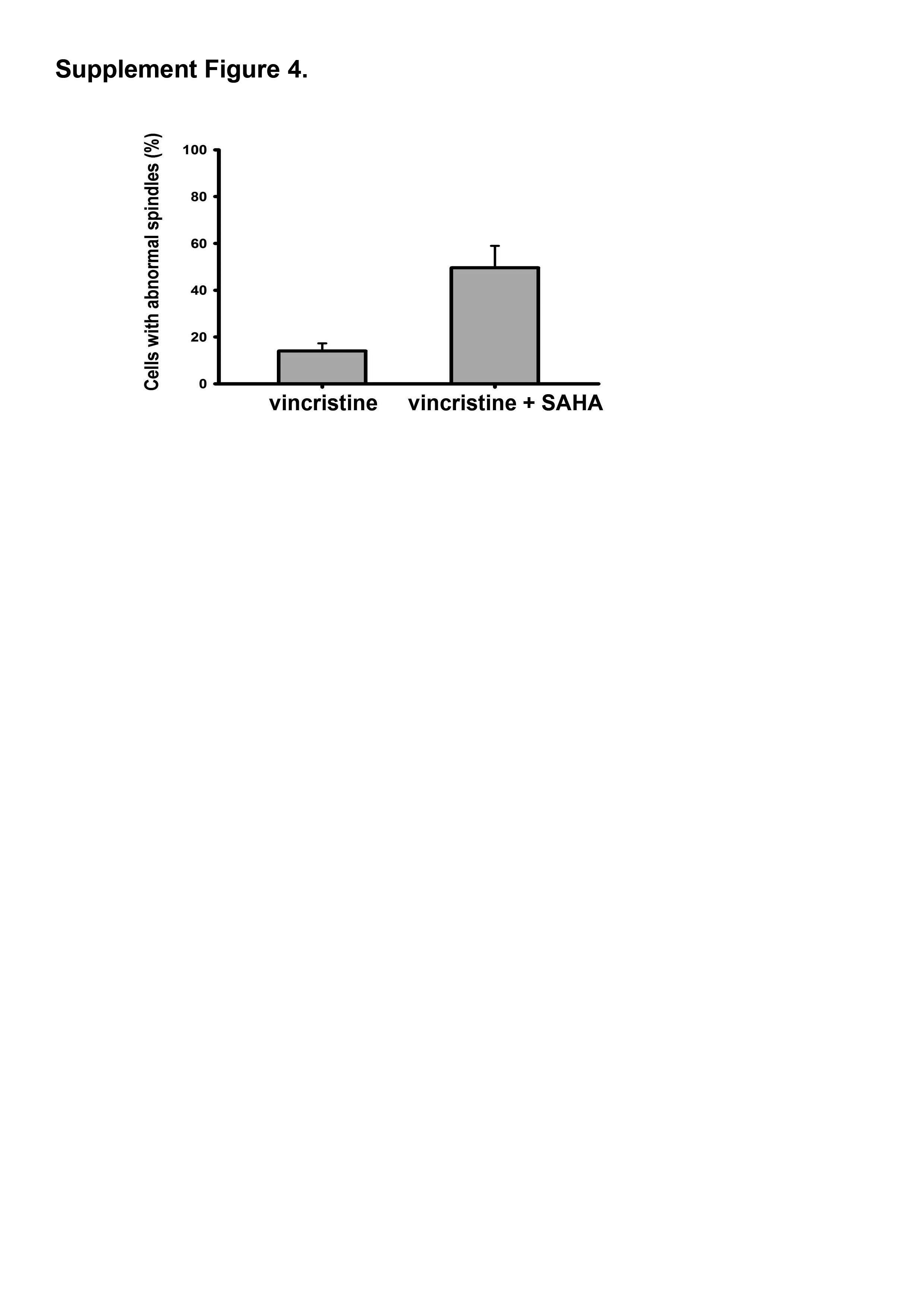

Supplement: Additional file 4: Figure S4. — The quantity of abnormal spindles in vincristine alone compared to combination with SAHA. MOLT-4 cells were treated with SAHA (0.5 μM) and vincristine alone (3 nM) or co-treatment for 24 h and then stained with β-tubulin and DAPI. The figure shows the quantity of abnormal spindles by the immunofluorescence images. [file 13045_2015_176_MOESM4_ESM.jpg]

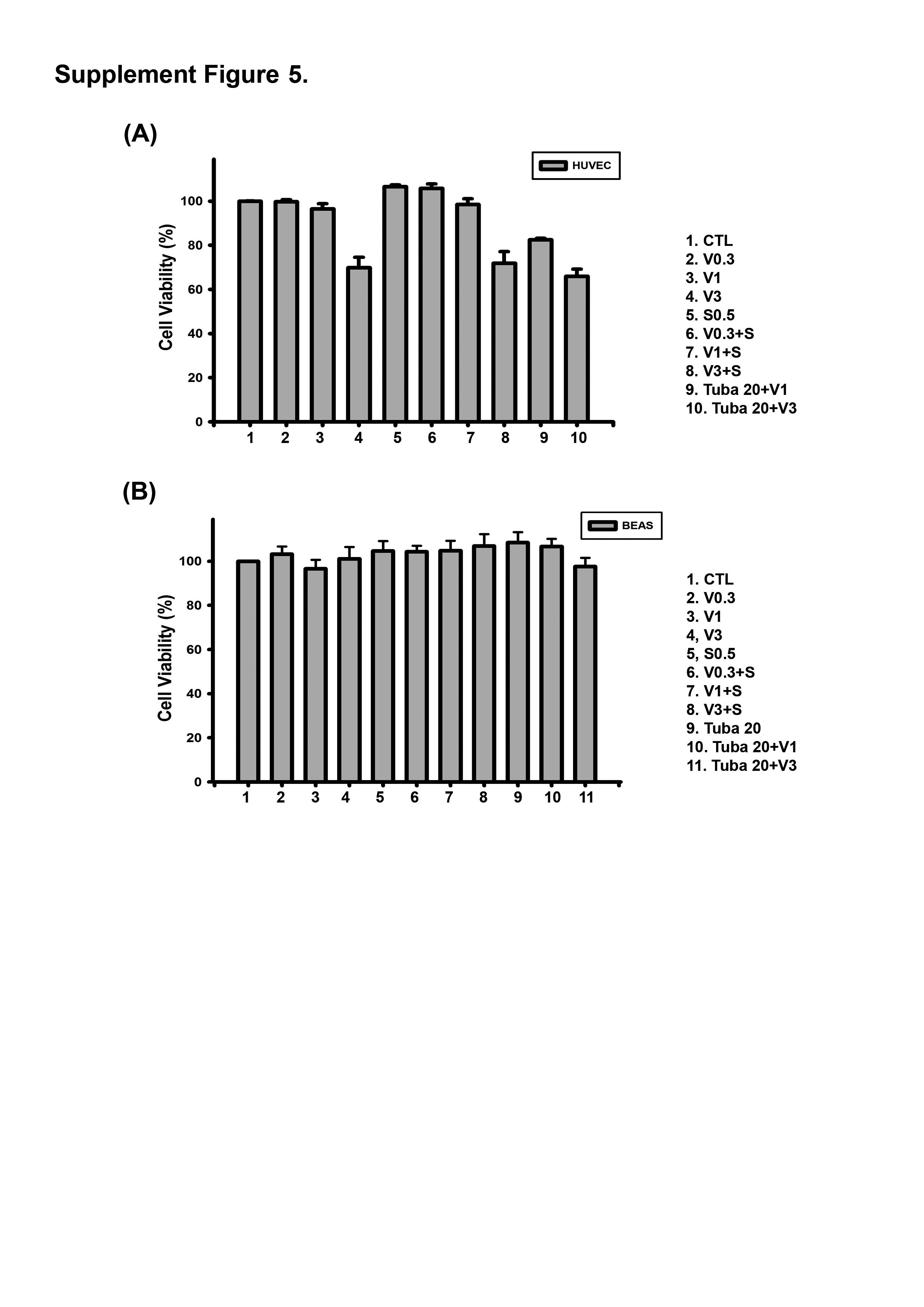

Supplement: Additional file 5: Figure S5. — The cell toxicity of normal cells after combination treatment. The cell viability (MTT assay) of (A) HUVEC (human umbilical vein endothelial cell) and (B) BEAS-2B (human bronchial epithelial cell) after co-treating with the indicated drugs for 48 h. [file 13045_2015_176_MOESM5_ESM.jpg]
